# On Being and Having: A Qualitative Study of Self-Perceptions in Bipolar Disorder

**DOI:** 10.1192/j.eurpsy.2025.1051

**Published:** 2025-08-26

**Authors:** A. L. Chang, M. Ponticiello, R. J. Chang, S. Mirza, A. Martin

**Affiliations:** 1 Yale School of Medicine; 2 Yale Child Study Center, New Haven, CT, United States

## Abstract

**Introduction:**

Bipolar disorder (BD) is a chronic and often severe mental illness. Yet despite the well-documented complexities in its diagnosis and treatment, little research has been dedicated to understanding the complex inner landscape experienced by those living with BD. Even as qualitative research has explored the lived experience of BD across a variety of perspectives, i.e., what BD *looks like*, there is a lack of research exploring what BD *means* to those living with the condition.

**Objectives:**

We aimed to understand how people with BD perceive their condition, construct the meaning of their illness, and view BD in relation to their sense of self.

**Methods:**

We conducted individual, semi-structured interviews with 20 adults with clinically stable BD. We coded the transcripts according to the principles of thematic analysis and analyzed the data using an interpretative phenomenological analysis approach.

**Results:**

We identified three overarching domains: (1) *Benefit or burden:* a dialectic through which participants weighed the valence of their illness over time; (2) *Self or other*: the internal or external locus through which they experienced BD; and (3) *From ineffability to meaning making:* the process of naming, understanding, and incorporating BD into their life’s whole. Within each domain, themes and subthemes outline nuanced and often conflicting perspectives of participants’ illness experiences.

**Conclusions:**

Our work provides a framework of three domains central to the inner reality of lived bipolar experience. Thoughtful understanding of patients’ experiences, perspectives, and desires within these three domains may aid clinicians and loved ones alike in more sensitively and effectively addressing the unique individual needs of those living with BD. By exploring patients’ perspectives in each of the three domains we identified, those caring for people with BD may be better positioned to help identify the inner work and practical interventions needed to achieve a rich, meaningful life with BD.

**Disclosure of Interest:**

None Declared

